# Rethinking the diagnosis of primary progressive aphasia: current challenges and future directions

**DOI:** 10.3389/fnagi.2025.1736855

**Published:** 2025-12-03

**Authors:** Joël Macoir, Monica Lavoie, Guillaume Duboisdindien, Robert Laforce

**Affiliations:** 1 Faculté de Médecine, École des Sciences de la Réadaptation, Université Laval, Québec, QC, Canada; 2 Centre de Recherche CERVO—Brain Research Centre, Québec, QC, Canada; 3 Chaire de Recherche sur les Aphasies Primaires Progressives – Fondation de la Famille Lemaire, Québec, QC, Canada; 4 Laboratoire LINC, UMR 1322 INSERM, UFR Sciences de la Santé, Université Marie and Louis Pasteur, Besançon, France; 5 Psychology and Cognitive Neuroscience Laboratory – PsyNCog, Université de Liège, Liège, Belgium; 6 Département des Sciences Neurologiques, Clinique Interdisciplinaire de Mémoire (CIME) du CHU de Québec, Québec, QC, Canada

**Keywords:** primary progressive aphasia, neurodegenerative diseases, diagnosis, language assessment, biomarkers, cultural considerations

## Abstract

Primary progressive aphasia (PPA) is a clinical syndrome characterized by the progressive decline of language abilities due to neurodegenerative disease. Accurate and timely diagnosis is critical for effective clinical management, patient support, and research participation. However, several diagnostic challenges persist, including limitations in current syndromic classifications, lack of linguistically and culturally standardized assessment tools, overlap with other neurocognitive disorders, heterogeneity in underlying pathology, limited biomarker specificity, and unequal access to specialized care. This article reviews key diagnostic barriers and proposes future directions to improve the clinical identification and classification of PPA, with an emphasis on cross-linguistic and cross-cultural considerations.

## 1 Introduction

Primary progressive aphasia (PPA) is a clinical syndrome characterized by the gradual and selective decline of language functions due to neurodegenerative disease. First described by Mesulam in the 1980s (Mesulam, 1982), PPA has since become a central model for studying the neural bases of language and their progressive disruption in neurocognitive disorders. Unlike post-stroke aphasia, which emerges suddenly following a vascular event, PPA evolves insidiously, placing unique demands on diagnosis, clinical management, and patient support (Marshall et al., 2018). Accurate and timely identification is critical not only for guiding intervention treatment and counseling but also for ensuring appropriate inclusion in research studies and clinical trials (Henry and Grasso, 2018; Mouton et al., 2022).

Over the past two decades, the field has advanced considerably in the clinical characterization of PPA. A breakthrough was the publication of consensus criteria by Gorno-Tempini et al. (2011), establishing three principal syndromic variants: the nonfluent/agrammatic (nfvPPA), the semantic (svPPA), and the logopenic (lvPPA). These criteria have improved consistency in diagnosis and facilitated research on disease mechanisms. However, while these criteria have structured the field, growing clinical experience has revealed important limitations. A substantial number of patients do not fit neatly into one of the three subtypes, instead showing atypical profiles (Botha et al., 2015; Tippett, 2020). In addition, longitudinal follow-up shows that while core language profiles remain relatively stable, most patients develop additional cognitive, behavioral, or motor features over time (de la Sablonnière et al., 2021; Foxe et al., 2022). This evolving clinical picture can obscure subtype distinctions and increase overlap with other neurodegenerative syndromes (Mesulam et al., 2022; Suárez-González et al., 2021).

Despite these advances, several diagnostic challenges remain, particularly regarding assessment tools, differential diagnosis, and neuropathological heterogeneity. First, reliable classification depends heavily on comprehensive language assessment, yet no universally consensual or standardized batteries exist across languages and clinical contexts. Moreover, many of the tools currently used in clinical and research settings were originally developed for post-stroke aphasia, which may limit their sensitivity, specificity, and ecological validity for capturing the progressive and variant-specific features of PPA (Clark et al., 2020; Henry and Grasso, 2018). Most available tools have also been developed and normed within English-speaking populations, limiting their applicability across diverse linguistic and cultural groups. Second, the clinical presentation of PPA often overlaps with other neurocognitive disorders, including Alzheimer’s disease (AD) (Rogalski et al., 2016), corticobasal syndrome (Ulugut et al., 2022), behavioral variant frontotemporal dementia (Rohrer and Warren, 2010), and mild cognitive impairment (Powell et al., 2016) with language deficits, thus complicating differential diagnosis. Third, the underlying neuropathology of PPA is heterogeneous: while semantic and nonfluent/agrammatic variants are more commonly linked to frontotemporal lobar degeneration (FTLD), the logopenic variant is most often associated with Alzheimer’s pathology (Bergeron et al., 2018), though exceptions abound (Montembeault et al., 2018; Spinelli et al., 2017). Currently available biomarkers, including cerebrospinal fluid assays and molecular imaging, improve diagnostic precision but remain insufficiently specific (Paraskevas et al., 2017; Santangelo et al., 2014) and unevenly accessible across healthcare systems.

Beyond these scientific and clinical challenges, systemic and equity-related issues further compromise timely diagnosis. Specialized memory and language clinics are unevenly distributed geographically, and access to expert evaluation is particularly limited in low-resource settings (Gallée et al., 2024). Cross-linguistic inequities also persist, as most of the diagnostic research and clinical protocols remain heavily skewed toward Western, educated, industrialized, rich, and democratic populations. These barriers collectively contribute to delayed or uncertain diagnoses, reduced patient and family support, and missed opportunities for therapeutic intervention and research participation.

Considering these limitations, this position paper aims to re-examine current diagnostic approaches to PPA. Specifically, it (i) reviews the main barriers to accurate and timely diagnosis, (ii) critically examines the adequacy of existing syndromic classifications and assessment methods, and (iii) outlines future directions for improving clinical practice. Particular attention is given to the pivotal role of speech-language pathologists (SLPs), who often serve as the first professionals to identify progressive language decline and initiate diagnostic pathways. Their expertise is essential not only for conducting sensitive assessments but also for advocating for patients in settings where access to neurology or neuroimaging may be limited. Special emphasis is also placed on cross-linguistic and cross-cultural perspectives, as equitable diagnostic strategies—implemented by well-trained SLPs worldwide—are essential for advancing both clinical care and scientific understanding.

To guide the reader, the article is structured as follows: Section 2 details the principal diagnostic barriers across five domains—from syndromic instability to inequitable access to expertise. In Section 3 evidence-based recommendations and future directions for clinical practice, education, and research are outlined. Section 4 concludes by summarizing key priorities for achieving more accurate, equitable, and globally relevant PPA diagnosis.

## 2 Key factors hindering early and accurate identification of PPA

### 2.1 Diagnostic framework and syndromic instability

The publication of the 2011 international consensus criteria by Gorno-Tempini and colleagues marked a critical advancement in the clinical conceptualization of PPA. This framework introduced a classification of PPA into three core clinical variants—nfvPPA, svPPA, and lvPPA, based on specific patterns of speech, language, and neuroimaging findings. These criteria have become widely adopted in research and clinical settings and have provided a much-needed structure for characterizing the linguistic features of progressive aphasia. The core features and main limitations of the 2011 criteria are summarized in Box 1.

BOX 1 **2011 Consensus criteria for primary progressive aphasia.**

Core variants and features

- nfvPPA: effortful, agrammatic speech; apraxia of speech common.
- svPPA: fluent speech with impaired word comprehension and loss of semantic knowledge.
- lvPPA: word-finding pauses; phonological errors during repetition.

Main strengths

- Established a unified, internationally accepted framework.
- Improved comparability across clinical and research settings.

Main limitations

- Frequent mixed or unclassifiable cases.
- Non-linguistic symptoms (behavioral, motor, cognitive) underrepresented.
- Cross-linguistic validity; psycholinguistic variability rarely addressed.
- Diagnostic boundaries often blurred by clinicopathological overlap.

lvPPA, logopenic variant of primary progressive aphasia; nfvPPA, nonfluent/agrammatic variant of primary progressive aphasia; svPPA, semantic variant of primary progressive aphasia.

However, many individuals with PPA present symptom profiles that do not align neatly with any single variant. So-called mixed or unclassifiable presentations are common, particularly in early disease stages, when deficits are subtle, or in more advanced stages, when deficits may be overlapping with other neurocognitive disorders. This clinical heterogeneity complicates confident classification (Mazzeo et al., 2024a; Utianski et al., 2019; Watanabe et al., 2024) and underscores the need for a more flexible, spectrum-informed framework that accommodates the graded nature of PPA presentation.

One important limitation of the current diagnostic criteria is their applicability to languages other than English. There is a growing body of literature showing that language-specific structural and lexical characteristics can influence PPA phenotypes (e.g., García et al., 2023; Mazzeo et al., 2024b). Therefore, diagnostic criteria should reflect these cross-linguistic and cross-cultural differences.

Moreover, the current criteria emphasize linguistic features while often neglecting broader cognitive, behavioral, or motor symptoms. For instance, patients with nfvPPA may show apraxia of speech, executive dysfunction (Illán-Gala et al., 2024; Macoir et al., 2017a) or parkinsonian signs (Henderson et al., 2024; Santos-Santos et al., 2016), whereas svPPA is frequently associated with behavioral changes (de la Sablonnière et al., 2021) that blur distinctions from the behavioral-variant frontotemporal dementia. Such overlaps, especially in the absence of biomarkers or longitudinal data, can hinder precise diagnosis and delay access to appropriate care. From a clinical management standpoint, diagnostic uncertainty affects prognosis, counseling and access to variant-specific interventions or research protocols. For SLPs, who often lead language assessments, accurate variant identification shapes treatment goals, tool selection, intervention strategies, and the needs for interdisciplinary collaboration. Yet in many non-specialist contexts, SLPs must navigate diagnostic ambiguity with limited standardized resources, increasing variability in care.

A further conceptual limitation is that the current PPA criteria describe deficits in terms of observable clinical manifestations rather than their underlying cognitive mechanisms. For example, the lvPPA is defined partly by impaired sentence repetition, yet the criteria do not specify that this deficit arises primarily from a phonological short-term memory disorder rather than from syntactic or articulatory difficulties. Similarly, agrammatism in nfvPPA or semantic degradation in svPPA may reflect disruptions in distinct cognitive processes that are not explicitly delineated in the diagnostic framework. By focusing on surface-level symptoms instead of the cognitive-functional origins of impairment, the existing criteria may obscure mechanistic distinctions that are essential for understanding, predicting, and targeting disease progression.

In sum, while the 2011 consensus criteria established an invaluable framework, real-world application reveals its limits. Overlapping, evolving, or atypical presentations call for greater diagnostic flexibility and the inclusion of non-linguistic features in variant definitions. Addressing this “syndromic instability” is crucial for timely and accurate diagnosis, equitable care, and the design of personalized interventions.

Importantly, syndromic instability may also reflect the underlying neuropathological heterogeneity of PPA. The same clinical variant can arise from distinct pathological processes, and conversely, identical pathologies can manifest through different linguistic profiles. This clinicopathological dissociation, developed further in Section 2.4, illustrates the need for diagnostic models that move beyond rigid categorical boundaries toward multidimensional, probabilistic frameworks that integrate linguistic, cognitive, and biological markers.

### 2.2 Limitations in language assessment tools

Despite advances in the clinical characterization of PPA, significant barriers persist in clinical practice when assessing language deficits. Chief among these is the limited availability of standardized, efficient, and culturally appropriate diagnostic tools. Most of the widely used batteries, such as the Boston Diagnostic Aphasia Examination (BDAE; Goodglass et al., 2001) in English, or the Batterie d’Évaluation cognitive du Langage (BECLA; Macoir et al., 2016) in French, were originally developed for stroke-induced aphasia and are not optimized for detecting the insidious, variant-specific deficits typical of PPA. They are often time-consuming, require specialized training, and may fail to capture the earliest manifestations of progressive language change.

A further challenge concerns the linguistic and cultural scope of available tools. Most have been created and validated in English-speaking, Western populations, and their direct use in other languages risks bias, reduced sensitivity, and misinterpretation. Tasks relying on culture-specific concepts, lexical frequencies, or morphosyntactic structures may either fail to detect comparable deficits or falsely suggest impairment, forcing clinicians in many regions to rely on non-standardized, informally adapted, or poorly validated instruments.

Orthographic transparency introduces additional complexity: opaque languages such as English or French tend to highlight semantic breakdowns in written tasks (e.g., surface dyslexia or agraphia in svPPA), whereas transparent orthographies, such as Spanish, Italian, or Turkish, may mask or attenuate these impairments unless assessments are carefully adapted to local grapheme–phoneme regularities.

Beyond orthography, core psycholinguistic variables, lexical frequency, word length, morphological complexity, imageability, phonological neighborhood density, and age of acquisition, strongly modulate performance in PPA (Bird et al., 2000; Vonk et al., 2019) but are insufficiently controlled for in many assessment tools. These features shape variant profiles but complicate cross-linguistic adaptation: low-frequency best reveal semantic loss in svPPA (Jefferies and Lambon Ralph, 2006), morphologically and syntactically complex structures expose deficits in nfvPPA (Wilson et al., 2010) and length-dependent repetition demands are particularly sensitive to phonological deficits in lvPPA (Lukic et al., 2019; Macoir et al., 2024). Because such variables differ across languages, assessments lacking matched stimuli or population-specific normative data risk producing misleading results. Box 2 lists key language and psycholinguistic factors that influence assessment accuracy and cross-linguistic comparability.

BOX 2 **Key linguistic and psycholinguistic variables affecting PPA diagnosis.**

Key task-level factors

- Lexical frequency: low-frequency words expose semantic loss.
- Age of acquisition/imageability: early, imageable words more resilient.
- Morphological/syntactic complexity: highlights agrammatism in nfvPPA.
- Phonological length/neighborhood density: strain phonological working memory in lvPPA.
- Orthographic transparency: irregular languages reveal surface dyslexia; transparent ones may conceal it.

Implications

- Careful control of psycholinguistic variables is crucial for valid cross-linguistic comparison.
- Test adaptation must ensure equitable sensitivity across languages and writing systems.

lvPPA, logopenic variant of primary progressive aphasia; nfvPPA, nonfluent/agrammatic variant of primary progressive aphasia.

Beyond linguistic and psycholinguistic considerations, standardized assessment tools themselves present intrinsic limitations increasingly discussed in recent psychometric literature (Swan et al., 2023; Youngstrom et al., 2017). Because normative tests rely on static group-based comparisons, they often fail to capture interindividual trajectories, compensatory mechanisms, or the ecological variability of communication—issues particularly salient in progressive and fluctuating conditions such as PPA. Rigid adherence to population norms may obscure clinically meaningful changes or produce false negatives when age, education, or cultural factors are poorly represented in reference samples. In PPA, these constraints are especially critical, as progressive decline and linguistic diversity challenge the very assumptions underlying standardization. This underscores the need for adaptive, process-oriented, and contextually grounded frameworks that complement norm-based testing and better reflect real-world communication.

Greater investment in culturally sensitive test development, robust norming, and psycholinguistic control is therefore essential for accurate and equitable diagnosis. These challenges are particularly critical for SLPs, who frequently conduct frontline evaluations without access to variant-specific protocols or norms. The absence of brief validated tools adapted to different languages and healthcare systems impedes early detection, longitudinal monitoring, and individualized intervention. Although tools such as the Mini Linguistic State Examination (MLSE; Patel et al., 2022), the Detection Test for Language Impairments in Adults and the Aged (DTLA; Macoir et al., 2017b) and the Progressive Aphasia Severity Scale (PASS; Sapolsky et al., 2014), show promise, their dissemination beyond research centers remains limited. Few existing tools offer multidimensional profiling in a time-efficient format.

A coordinated international effort is urgently needed to design and share brief, psychometrically robust, and culturally adaptable assessments. Such tools would not only enhance early diagnosis and treatment planning but also promote equity and comparability across populations. Equally crucial, future research should emphasize clinical approaches that capture individual trajectories, within-person variability, and the real-world functional impact of communication impairments. This requires greater openness to criterion-referenced and process-oriented methods, including descriptive analyses and language-sample–based assessments, which can track progressive change and compensatory adaptation more sensitively than norm-based tests alone. Recent longitudinal and speech-analysis studies support this need for trajectory-sensitive, process-oriented approaches that extend beyond static norm-based tools (Ash et al., 2019; Yeung et al., 2021; Gallée et al., 2023). Combining standardized and individualized approaches represents a promising path toward more ecological, person-centered evaluation of language in neurodegenerative conditions such as PPA.

### 2.3 Differential diagnosis

Distinguishing PPA from other neurocognitive and psychiatric conditions remains a central clinical challenge. At initial presentation, language impairments may be subtle, variable, or overshadowed by behavioral and emotional symptoms (Marshall et al., 2018; Ruksenaite et al., 2021), leading to misdiagnosis and delayed intervention. Box 3 provides a concise overview contrasting each PPA variant with its closest diagnostic look-alikes.

BOX 3 **Differential diagnosis at a glance.**

lvPPA vs. Alzheimer’s disease

- Shared: word-finding pauses, anomia, working-memory deficits.
- Distinguishing lvPPA: speech remains grammatical; comprehension preserved; disproportionate phonological errors and repetition deficits.
- Distinguishing AD: early episodic memory loss, visuospatial impairment, and broader cognitive decline.

svPPA vs. behavioral-variant frontotemporal dementia

- Shared: social–emotional and behavioral change (rigidity, loss of empathy).
- Distinguishing svPPA: profound loss of word meaning and surface dyslexia/agraphia.
- Distinguishing bvFTD: preserved naming and comprehension; prominent executive impairment and behavioral changes.

nfvPPA vs. PPAOS

- Shared: speech effort, motor symptoms, parkinsonism.
- Distinguishing nfvPPA: agrammatism and impaired sentence comprehension.
- Distinguishing PPAOS / PSP / CBS: speech sound distortions or motor-planning deficits predominate; syntax often preserved early.

Clinical note

Accurate differentiation requires comprehensive language assessment, longitudinal observation, and imaging or biomarker confirmation when available.

bvFTD, behavioral-variant frontotemporal dementia; CBS, Corticobasal syndrome; lvPPA, logopenic variant of primary progressive aphasia; nfvPPA, nonfluent/agrammatic variant of primary progressive aphasia; PPAOS, primary progressive apraxia of speech; PSP, progressive supranuclear palsy; svPPA, semantic variant of primary progressive aphasia.

The lvPPA exemplifies this difficulty. Patients exhibit word-finding pauses and phonological errors (Gorno-Tempini et al., 2011) that can resemble the anomia and working-memory limitations of early AD (Huntley and Howard, 2010; Pistono et al., 2019). Without detailed linguistic assessment, lvPPA can be mistaken for amnestic AD, a clinically consequential error, as the two conditions share identical pharmacological treatment yet differ in symptom trajectories, progression patterns, and support needs. Diagnostic confusion may also arise between lvPPA and nfvPPA, particularly early in the disease, when both may present with speech that appears slowed or interrupted by pauses. However, in lvPPA, these pauses reflect word-finding difficulty and reduced phonological short-term memory, whereas in nfvPPA they result from motor speech impairment and agrammatism (Lukic et al., 2019; Macoir et al., 2021).

svPPA presents another diagnostic pitfall. Alongside the progressive erosion of conceptual knowledge and word meaning, patients frequently develop behavioral and socio-emotional changes such as loss of empathy, rigid or compulsive behaviors, and altered dietary preferences (Foxe et al., 2022; Roy et al., 2023). These features overlap with behavioral variant frontotemporal dementia (Kamath et al., 2019; Ramanan et al., 2022) and may also be misinterpreted as depression (Szymkowicz et al., 2023) in non-specialist settings, delaying appropriate recognition of the underlying language disorder.

In nfvPPA, diagnosis can be challenging when motor-speech impairments—particularly apraxia of speech—predominate particularly apraxia of speech. Such cases may first be labeled primary progressive apraxia of speech (PPAOS) (Duffy et al., 2021) or mistaken for movement disorders such as progressive supranuclear palsy or corticobasal syndrome (Krzosek et al., 2022; Peterson et al., 2021) which may later co-occur. Emerging evidence suggests that nfvPPA and PPAOS may lie along a shared continuum, displaying overlapping features yet maintaining distinct onset profiles and evolutionary trajectories (Duffy et al., 2021; Garcia-Guaqueta et al., 2024; Illán-Gala et al., 2024; Lorca-Puls et al., 2023).

Accurate differential diagnosis therefore requires the synthesis of detailed language testing, comprehensive neuropsychological profiling, and longitudinal observation. Although follow-up can eventually clarify uncertain cases, delays in classification postpone access to appropriate counseling and therapy. Moreover, as disease progresses and additional cognitive, behavioral and motor deficits emerge, distinguishing between the three PPA variants becomes increasingly challenging, reinforcing the need for timely, early-stage identification. Early, accurate identification, based on standardized linguistic assessment and diverse interpretive approaches (norm-referenced, criterion-referenced, dynamic, and descriptive) and informed by multidisciplinary input, is essential both for optimal care and for inclusion in research trials targeting variant-specific mechanisms.

### 2.4 Neuropathological and biomarker challenges

While careful clinical assessment is essential for distinguishing PPA from other neurocognitive and psychiatric conditions, even accurate syndromic classification provides only a partial picture. One of the defining complexities of PPA One of the defining complexities of PPA lies in its neuropathological heterogeneity. Unlike post-stroke aphasia, which is tied to a focal vascular lesion, PPA can result from diverse underlying pathologies, most commonly FTLD or AD (Gorno-Tempini et al., 2011; Mesulam et al., 2014a; Mesulam et al., 2014b). Crucially, the relationship between clinical presentation and underlying disease is not one-to-one: the same PPA variant may arise from different biological processes. For example, nonfluent/agrammatic PPA may be caused by either FTLD-tau or FTLD-TDP (Grossman, 2010; Rusina et al., 2022), while a single pathology such as AD can manifest through multiple phenotypes, most often the lvPPA but occasionally others (Mesulam et al., 2014a; Mesulam et al., 2014b). This clinicopathological dissociation complicates prognosis, counseling, and efforts to develop targeted interventions. Current and future biomarker strategies are overviewed in Box 4.

BOX 4 **Biomarkers and multimodal approaches.**

Established tools (utility)

- CSF Aβ/tau: supports AD-related lvPPA; helpful in atypical presentations.
- Amyloid / Tau PET: increases etiological certainty (AD vs. FTLD) when clinical picture is ambiguous.
- MRI / FDG-PET: characteristic but non-pathognomonic patterns (svPPA anterior temporal; nfvPPA IFG/insula; lvPPA temporoparietal).

Key limitations

- Clinicopathological dissociation persists (same phenotype, different pathology; same pathology, different phenotype).
- Access & cost: PET scarce outside tertiary centers; lumbar puncture underused; interpretation expertise uneven.
- No single biomarker reliably distinguishes variants or predicts progression at the individual level.

Emerging directions

- Blood-based biomarkers (e.g., plasma p-tau, NfL): less invasive, more scalable complements to CSF/PET.
- Digital/linguistic markers: connected speech, lexical–syntactic metrics, prosody, and error profiles as candidate endpoints.
- Multimodal ML models: integrate language features + MRI/FDG/PET + fluid biomarkers to improve individualized pathology prediction.
- AI-driven analytics: deep-learning and natural-language-processing approaches automatically extract acoustic, lexical, and syntactic features from speech samples; combine these with imaging or biomarker data to enhance early classification, subtype discrimination, and longitudinal monitoring

Practice guidance

- Use a stepwise approach: detailed language assessment → MRI/FDG → CSF/PET or blood biomarkers when needed.
- Interpret results probabilistically, alongside clinical and neurolinguistic data.
- Prioritize equity: tele-evaluation, shared protocols, and regional referral pathways to reduce access gaps.

AD, Alzheimer’s disease; AI, artificial intelligence; Aβ, amyloid-beta; CSF, cerebrospinal fluid; FDG, fluorodeoxyglucose; FTLD, frontotemporal lobar degeneration; IFG, inferior frontal gyrus; lvPPA, logopenic variant of primary progressive aphasia; MRI, magnetic resonance imaging; NfL, neurofilament light chain; nfvPPA, nonfluent/agrammatic variant of primary progressive aphasia; PET, positron emission tomography; p-tau, phosphorylated tau protein; svPPA, semantic variant of primary progressive aphasia.

Broad associations between clinical subtypes and pathology have been described but remain imperfect. svPPA is most consistently linked to FTLD-TDP type C, although a minority of cases show tau pathology (Ma et al., 2025; Mesulam et al., 2014a; Mesulam et al., 2014b). nfvPPA is heterogeneous, with underlying pathology divided between FTLD-tau (including progressive supranuclear palsy and corticobasal syndrome) and FTLD-TDP (Giannini et al., 2019; Montembeault et al., 2018). lvPPA is most often associated with AD pathology (Bergeron et al., 2018), but cases related to FTLD have also been reported (Mesulam et al., 2022; Shir et al., 2024), and recent imaging evidence suggests that Lewy body disease may occasionally contribute to amyloid-negative presentations (Kang et al., 2025). These probabilistic can assist clinical reasoning but do not provide diagnostic certainty at the individual level.

Structural MRI and FDG-PET reveal characteristic but overlapping patterns of atrophy or hypometabolism (e.g., anterior temporal for svPPA, left inferior frontal/insula for nfvPPA, and left temporoparietal for lvPPA) (Mirbod et al., 2024). However, these imaging signatures are not pathognomonic: while anterior temporal atrophy is typical of svPPA, it is also observed in right-anterior-temporal–predominant FTD, a syndrome clinically distinguishable from svPPA (Eldaief et al., 2023; Ulugut et al., 2024). Likewise, temporoparietal atrophy, although considered a hallmark of lvPPA, is also frequently observed in atypical presentations of AD without aphasia, such as posterior cortical atrophy or dysexecutive variants, limiting its diagnostic specificity (Townley et al., 2020; Whitwell et al., 2018).

Despite their diagnostic value, advanced biomarkers remain unevenly available across clinical settings. PET imaging is costly and often restricted to research centers (Rabinovici et al., 2025). Lumbar puncture is not consistently available and is often perceived as an invasive procedure. Moreover, many clinicians remain insufficiently trained to interpret CSF profiles or unconvinced of the added clinical value of biomarker confirmation (Bouwman et al., 2022; Hampel et al., 2022). Access is especially limited in low- and middle-income countries, where uneven diffusion of biomarker technology compounds existing cross-linguistic and cross-cultural inequities in diagnosis and research participation (Babulal et al., 2024; McGlinchey et al., 2024). Importantly, these biomarkers are most sensitive to AD pathology and therefore particularly useful for identifying AD-related lvPPA (Iaccarino et al., 2023; Rullmann et al., 2025).

Given these challenges, no single biomarker currently provides a reliable and universally applicable method for distinguishing among PPA variants or predicting progression. Future progress will likely depend on multimodal approaches that integrate clinical, neuropsychological, neuroimaging, and biological data. Promising developments include blood-based biomarkers such as plasma phosphorylated tau and neurofilament light chain, which may broaden access by reducing cost and invasiveness (Grande et al., 2025; Liampas et al., 2024). Machine learning models that combine multimodal data, including language performance, structural and functional imaging, and biomarker measures (Rezaii et al., 2024; Tafuri et al., 2024), show growing potential for improving individual-level prediction of underlying pathology (for a review, see Macoir et al., 2025).

### 2.5 Limited access to expertise and training

The accurate diagnosis of PPA often depends on coordinated input from a multidisciplinary team that typically includes neurologists, nurses, SLPs, and neuropsychologists. Such collaborative expertise is most often found in tertiary memory or language clinics, yet access to these centers is uneven both within and across countries (Gallée et al., 2024; Gulline et al., 2025). Patients in rural areas or low-resource regions frequently encounter substantial delays before being referred to specialized services, if referral occurs at all (Villaseñor and Saidi, 2024). Box 5 highlights the main challenges and priority action related to inequitable access to clinical expertise and training in PPA diagnosis.

BOX 5 **Limited access to expertise and training.**

Key challenges

- Specialized PPA clinics concentrated in large urban or academic centers.
- Delays or absence of referral for patients in rural or low-resource regions.
- Minimal PPA coverage in medical, geriatric, and SLP curricula.
- Continuing-education opportunities and standardized protocols remain scarce.

Consequences

- Frequent under- or misdiagnosis, leading to late intervention and exclusion from research.
- Unequal access to counseling, therapy, and multidisciplinary care.

Action priorities

- Integrate PPA content into medical and SLP training programs.
- Expand tele-education and international mentorship networks.
- Recognize diagnostic equity as a public-health goal supported by policy and funding initiatives.

PPA, primary progressive aphasia; SLP, speech-language pathology.

A further challenge lies in the limited awareness and training of clinicians and allied health professionals. Surveys and practice reports indicate that many neurologists, geriatricians, and SLPs receive little formal instruction on recognizing the early language-led presentations of neurodegenerative disease. This lack of formation is particularly pronounced for PPA, which remains underrepresented in most medical and speech-language pathology curricula (Battista et al., 2023; Gallée et al., 2024). Training programs and diagnostic pathways continue to emphasize amnestic AD, predisposing clinicians to overlook or misinterpret progressive language symptoms (Giebel et al., 2024). As a result, early manifestations of PPA may be attributed to normal aging, psychiatric conditions such as depression or anxiety (Mulder-Heijstra et al., 2022), or classified under nonspecific dementia labels, preventing patients from receiving variant-specific management and support (Hall et al., 2013).

These knowledge gaps have direct consequences for patient care. Underdiagnosis or misdiagnosis leads to delays in counseling, limits opportunities for time –sensitive speech-language intervention and impedes access to research protocols and clinical trials (Battista et al., 2023; Mouton et al., 2022). Even when PPA is recognized, variability in clinical experience means that care pathways can differ considerably from one region to another, with some patients receiving comprehensive multidisciplinary support and others relying on informal assessments or generic dementia management strategies (Gallée et al., 2024).

Addressing this inequity requires both systemic and educational responses. Integrating PPA more thoroughly into medical and speech-language pathology curricula, disseminating consensus diagnostic guidelines, and developing accessible, standardized brief assessment protocols could substantially improve early recognition. Telemedicine (Yi et al., 2021) and international training initiatives (Gallée et al., 2024) may also help extend specialist expertise to regions without tertiary centers. Without such measures, disparities in professional training and clinical access will continue to widen inequities in PPA diagnosis and care.

The clinical consequences of these limitations are far-reaching. Delayed or inaccurate diagnosis often results in missed opportunities for early intervention and communication-focused therapy, at a stage when compensatory tools and strategies, relying on preserved capacities, are most effective (Marshall et al., 2018). Patients and families may not receive timely counseling, support for daily communication, or guidance on planning for disease progression (Shibata et al., 2024). Inaccurate or nonspecific diagnoses can also exclude individuals from clinical research or variant-targeted treatment trials. In contrast, early recognition enables clinicians to implement tailored speech-language interventions, educate caregivers, and facilitate access to appropriate medical and social resources, thereby enhancing quality of life and ensuring greater continuity of care.

## 3 Recommendations and future directions

Improving the diagnosis and management of PPA requires an integrated strategy spanning clinical, educational, and research domains. The aim is to refine diagnostic accuracy while ensuring equitable access to expertise, culturally valid tools, and evidence-based interventions worldwide. Box 6 synthesizes the key recommendations and priorities for future action.

BOX 6 **Future directions.**

Immediate priorities

- Update diagnostic frameworks to reflect clinical heterogeneity and biological diversity.
- Develop standardized, cross-linguistic tools ensuring equitable assessment across cultures.
- Integrate multimodal and machine-learning approaches for precision and prognosis.

Structural goals

- Invest in professional training and tele-education to democratize expertise globally.
- Implement early, variant-specific interventions through coordinated care networks.

Long-term vision

- Build shared international databases and open-science infrastructures for reproducible research.
- Foster inclusive, culturally sensitive collaborations so that improving PPA diagnosis also improves the lived experience of those affected.

PPA, primary progressive aphasia.

### 3.1 Expanding professional education and awareness

Earlier and more reliable recognition of PPA depends on stronger and more widespread professional training. Instruction on language-led neurodegenerative presentations should be incorporated into neurology, geriatrics, and speech-language-pathology curricula to correct the prevailing bias toward amnestic AD and help clinicians detect subtle, language-dominant syndromes. Continuing-education programs, teleconsultation networks, and online modules can disseminate expertise to clinicians in low-resource or rural areas, thus reducing geographic inequities. Broader awareness of variant-specific features will minimize misclassification and improve referrals. Professional associations and health ministries should support certification or microcredential pathways focused on PPA, promoting multidisciplinary collaboration among neurologists, neuropsychologists, and SLPs to embed PPA within standard dementia-care frameworks.

### 3.2 Developing and disseminating cross-linguistic and culturally adapted assessments

Global equity requires standardized, linguistically valid tools reflecting structural and orthographic differences across languages. International collaborations should prioritize the adaptation and validation of brief screening measures, the creation of robust normative datasets, and the integration of psycholinguistic controls in test design. Open-access repositories of multilingual stimuli, scoring guides, and normative databases—modeled after initiatives in stroke aphasia or neuropsychology—would accelerate progress and prevent duplication. Harmonized cross-linguistic datasets will enhance generalizability and support robust meta-analyses and universal diagnostic metrics.

### 3.3 Revisiting diagnostic frameworks and consensus criteria

More than a decade after publication, the 2011 international consensus criteria (Gorno-Tempini et al., 2011) remain foundational but increasingly outdated. Mixed or atypical profiles, syndromic instability, and clinicopathological dissociation justify an updated, more flexible model integrating linguistic, cognitive, behavioral, imaging, and biomarker indicators. A revised consensus should: (a) define core and ancillary features along continua rather than categorical cut-offs; (b) incorporate quantifiable psycholinguistic metrics (e.g., lexical diversity, syntactic complexity); (c) recommend standardized brief core batteries; and (d) include updated biomarker-supported diagnostic tiers analogous to Alzheimer’s frameworks.

### 3.4 Integrating multimodal diagnostic data

Future diagnostic frameworks should combine clinical, neuropsychological, linguistic, imaging, and biological data into coherent into coherent multimodal pipelines. Blood-based biomarkers such as plasma phosphorylated tau and neurofilament light chain offer less-invasive complements to CSF and PET studies (Grande et al., 2025; Liampas et al., 2024). Integrating these markers with digital linguistic features and advanced imaging indices could improve individualized prediction of underlying pathology. Machine-learning approaches merging linguistic, neuroimaging, and biomarker inputs show strong potential for variant classification and disease monitoring (Rezaii et al., 2024; Tafuri et al., 2024), but clinical translation requires transparent algorithms, shared datasets, and standardized interpretive guidelines. Cross-institutional collaboration and open-science practices, including data sharing, preregistration, and reproducible pipelines, will be essential for validation and bias reduction.

### 3.5 Implementing early, evidence-based interventions

Early, accurate diagnosis must guide personalized intervention. PPA patients should access evidence-based therapies tailored to each variant’s linguistic and cognitive profile. Priorities include (a) compensatory communication strategies (Cadório and Vieira, 2025); (b) variant-specific approaches such as conversational coaching for lvPPA or lexical-retrieval treatment for svPPA (Pagnoni et al., 2021; Simic et al., 2025); and (c) caregiver training and counseling to foster supportive communication environments (Wong et al., 2025). Integrated care pathways linking neurology, speech-language pathology, neuropsychology, and social work should ensure continuity. Recognizing people with PPA as a target population for early intervention would legitimize these services and facilitate reimbursement. Early identification improves outcomes and expands access to clinical and disease-modifying trials.

### 3.6 Strengthening international collaboration and research equity

Global collaboration is essential to accelerate progress. Shared behavioral, imaging, and biomarker databases will improve reproducibility and cross-validation of diagnostic models. Multicenter studies must include linguistically and culturally diverse populations avoiding the historical over-representation of English-speaking cohorts. Longitudinal designs tracking progression across variants are needed to refine prognostic markers and treatment timing. Open-science frameworks, preregistration, transparent data curation, and FAIR-compliant repositories, will ensure that datasets are globally accessible and ethically shared. Such collaboration can transform PPA diagnosis from a fragmented field into a cohesive, internationally integrated enterprise.

## 4 Conclusion

PPA remains a poorly recognized and diagnostically challenging neurodegenerative syndrome, particularly in low-resource contexts, where awareness, specialized expertise, and access to appropriate assessment tools remain limited. Accurate and equitable diagnosis demands not only better instruments but also systemic efforts to strengthen education, refine policy, and enhance interdisciplinary coordination.

Key priorities include: (1) revising diagnostic frameworks to reflect clinical heterogeneity and biological diversity; (2) developing standardized, cross-linguistic assessments tools to support equitable global application; (3) integrating multimodal and machine-learning approaches to improve diagnostic precision and prognostic accuracy; (4) expanding professional training and tele-education pathways to democratize expertise; and (5) implementing early, variant-specific interventions within coordinated care networks.

Together, these measures could transform PPA from a niche clinical entity into a widely recognized and proactively managed condition, enabling earlier and more consistent support for patients and families. Sustained international collaboration and a commitment to open, equitable, and culturally sensitive science will be essential. Ultimately, the goal is not only to refine PPA classification but also to improve the lived experience and quality of life of those affected.

## Data Availability

The original contributions presented in the study are included in the article/supplementary material, further inquiries can be directed to the corresponding author.
